# Metastasis-associated DNA methylation of the lncRNAs *LINC01354* and *LINC01510* in clear cell renal cell carcinoma

**DOI:** 10.1186/s13148-026-02252-4

**Published:** 2026-10-13

**Authors:** Pouriya Faraj Tabrizi, Inga Mareike Schimansky, Inga Peters, Mohammed Rafea Kanaan, Jörg Hennenlotter, Andre Franke, Hossein Tezval, Markus Antonius Kuczyk, Jürgen Serth

**Affiliations:** 1 Department of Urology and Urological Oncology, Hannover Medical School, 30625 Hannover, Germany; 2 Department of Hematology, Hemostaseology, Oncology and Stem Cell Transplantation, Hannover Medical School, 30625 Hannover, Germany; 3 Department of Urology, Krankenhaus Nordwest, 60488 Frankfurt, Germany; 4 Department of Urology, Eberhard Karls University Tübingen, 72076 Tübingen, Germany; 5 Institute of Clinical Molecular Biology, Kiel University, 24105 Kiel, Germany

**Keywords:** Renal cell carcinoma, Metastasis, DNA methylation, Epigenetics, Long non-coding RNA

## Abstract

**Background:**

Long non-coding RNAs (lncRNAs) regulate multiple cellular processes and are involved in the development and progression of human cancers, including renal cell carcinoma (RCC).

**Methods:**

Genome-wide DNA methylation profiling using Illumina EPIC arrays was performed to identify differentially methylated regions (DMR) associated with metastatic RCC. Candidate loci within *LINC01354* and *LINC01510* were validated by pyrosequencing in 145 primary RCC tissues, 124 paired tumor-adjacent normal tissues (adNs), and 194 metastatic tissue samples obtained from 101 patients with metastatic RCC. In addition, data from The Cancer Genome Atlas Kidney Renal Clear Cell Carcinoma Collection (TCGA-KIRC) were analyzed in silico to assess the clinical relevance of DNA methylation (DNAm) and its association with gene expression.

**Results:**

Hypermethylation across all analyzed CpG sites at both loci was associated with high-stage (≥ T3; *p* = 0.001) and high-grade (≥ G3; *p* = 0.003) RCC and was markedly increased in metastatic tissues compared with localized primary tumors (*p* < 0.001). In addition, primary tumors from patients with distant metastases (M+) showed significantly higher methylation at PS_307-CG1 within *LINC01510* than tumors from patients without distant metastases (M0; *p* = 0.031). Analyses of TCGA-KIRC data confirmed associations of increased methylation with adverse clinicopathological features and reduced gene expression, supporting a potential role for epigenetic silencing.

**Conclusion:**

Our data provide further insight into the epigenetic alterations associated with RCC progression by demonstrating tumor- and metastasis-associated hypermethylation at the *LINC01354* and *LINC01510* loci. The association of hypermethylation with advanced disease and metastatic tissues, together with the observed inverse relationship between methylation and expression, suggests that epigenetic inactivation of these lncRNAs may contribute to RCC progression. DNAm at these loci may therefore represent a potential biomarker of aggressive disease.

**Supplementary Information:**

The online version contains supplementary material available at https://doi.org/10.1186/s13148-026-02252-4.

## Introduction

Renal cell carcinoma (RCC) is among the ten most common cancers worldwide, and its incidence continues to increase [1]. Clear cell renal cell carcinoma (ccRCC) is the most common histological subtype, accounting for approximately 75% of all RCC cases [2, 3]. The increasing use of abdominal imaging has changed the epidemiology of RCC, with a growing proportion of tumors being detected incidentally at earlier stages [4].

Novel systemic therapies, particularly immune checkpoint inhibitors such as the PD-1 inhibitors pembrolizumab and nivolumab, as well as tyrosine kinase inhibitors such as axitinib and lenvatinib, have substantially changed the management and outcomes of advanced RCC over the past decade. Recently, targeting the hypoxia-inducible factor 2 alpha (HIF-2α) pathway has emerged as an additional therapeutic approach. The HIF-2α inhibitor belzutifan demonstrated significant improvements in progression-free survival and objective response rate compared with everolimus in patients with advanced RCC who had previously received immune checkpoint inhibitors and antiangiogenic therapy [5]. Belzutifan has also demonstrated an objective response rate of 67% after a median follow-up of 41 months in patients with Von Hippel-Lindau (VHL) disease-associated RCC [6]. More recently, the combination of pembrolizumab and belzutifan demonstrated improved disease-free survival compared with pembrolizumab monotherapy in patients with increased risk for recurrence after nephrectomy [7]. However, validated predictive biomarkers for individualized treatment selection remain lacking. This limitation may contribute to the persistently unfavorable outcomes of patients with advanced disease [8, 9]. More than 25% of patients with RCC present with metastatic disease at initial diagnosis, emphasizing the need to identify clinically relevant biomarkers and targetable molecular pathways [4].

The identification of novel biomarkers associated with individual tumor aggressiveness may provide a basis for functional studies and the development of personalized therapeutic strategies. However, validated biomarker panels for predicting metastatic progression in RCC remain limited [10].

At the molecular level, ccRCC development is characterized by genetic and epigenetic alterations. Alteration of the *VHL* tumor suppressor gene, located on chromosome 3p, represents a main driver of ccRCC development. The loss of the protein function through genetic or epigenetic alterations leads to activation of HIF signaling, thereby promoting angiogenesis and cellular proliferation [11]. In addition, other tumor suppressor genes located on chromosome 3p and frequently altered in ccRCC, *PBRM1*, *BAP1* and *SETD2,* are involved in chromatin regulation and contribute to tumor development and progression [12–17]. This molecular heterogeneity emphasizes the relevance of epigenetic dysregulation in ccRCC and supports the investigation of DNA methylation (DNAm) as one of several potential biomarkers.

We recently described a renal metastasis-associated methylation signature (RMAMS) as a potential DNAm-based approach for detecting and predicting metastatic RCC (mRCC) [18]. We also identified hypermethylation of the long non-coding RNA (lncRNA) genes *LINC00403* and *LINC00404* in metastatic primary RCC and RCC metastases, supporting their potential inclusion in metastasis-associated biomarker panels [19].

LncRNAs, together with microRNAs and pseudogene-derived transcripts, regulate cellular processes involved in cancer development and progression, including proliferation, invasion, and metastasis [20–22].

In RCC, altered expression of lncRNAs and pseudogene-derived transcripts has been associated with epigenetic dysregulation, including hypermethylation or hypomethylation of non-coding RNA loci, and may affect oncogenic signaling pathways [20, 23–25]. These findings highlight the potential role of lncRNAs in metastatic progression across different cancer types and their possible prognostic relevance [26].

*LINC01354*, located on chromosome 1, has been reported to exert oncogenic functions in several tumor entities. Altered *LINC01354* expression has been associated with disease progression in gastric, colorectal, and lung cancers as well as osteosarcoma [27].

*LINC01354* interacts with microRNAs and RNA-binding proteins and may function as a competing endogenous RNA (ceRNA) by sequestering cancer-related microRNAs. Through these interactions, *LINC01354* has been implicated in the dysregulation of oncogenic pathways, including Wnt/β-catenin signaling, and in the promotion of proliferation, invasion, and epithelial-mesenchymal transition [27]. However, DNAm changes at the *LINC01354* locus have not yet been described in RCC, and data on *LINC01354* methylation in other human cancers remain limited. Analyses of The Cancer Genome Atlas (TCGA) identified *LINC01354* as a potential prognostic marker in head and neck squamous cell carcinoma [28].

A TCGA-based pan-cancer analysis further suggested that *LINC01354* is subject to DNAm-associated epigenetic regulation in several cancer types. In lung adenocarcinoma, *LINC01354* was proposed to have a tumor-suppressive role, with methylation-associated silencing being linked to an unfavorable prognosis [29].

*LINC01510*, also known as *COMETT* (cytosolic oncogenic mRNA antisense to MET transcript), is located on chromosome 7 (Supplemental Fig. 1) [30]. It has been implicated in several cancer types, including RCC, colorectal cancer, lung cancer, and papillary thyroid cancer, although its expression and biological effects appear to be context dependent [31–35].

**Fig. 1:**
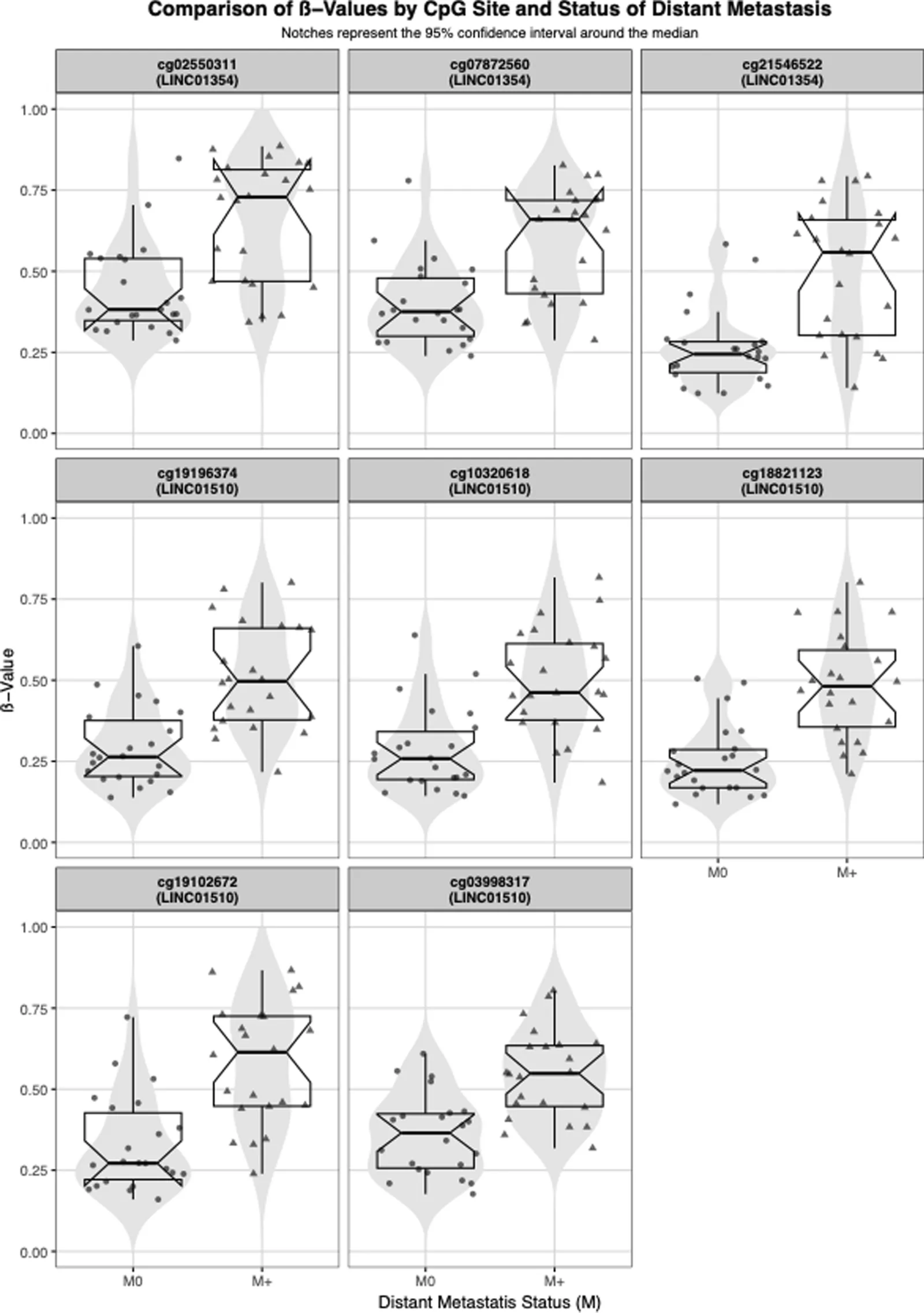
Distribution of DNA methylation β-values of candidate CpG sites following the DNA profiling discovery study comparing M0 and M+ primary RCC tumors. β-values reflect the proportion of methylation at each CpG site (range: 0–1). Boxplots represent median and interquartile range; notches are indicative of the 95% confidence interval. Shadowed areas indicate the density distribution of β-values

*LINC01510* has been reported to regulate cell proliferation, cell-cycle progression, invasion, and migration through several molecular pathways, including MET signaling and the miR-339-5p/CDK14 and BCL-2 axes. It may also act as a transcriptional enhancer and is itself subject to epigenetic regulation [32, 36, 37].

In non-small cell lung cancer (NSCLC), the histone methyltransferase KMT5C represses *LINC01510* through H4K20me3. Loss of KMT5C results in increased *LINC01510* and *MET* expression and promotes resistance to EGFR inhibitors [37]. Whereas increased *LINC01510* expression has been associated with an unfavorable prognosis in NSCLC and colorectal cancer [32, 34], reduced expression has been reported in RCC and was likewise associated with poor clinical outcomes [31]. *LINC01510* was also identified as part of a lncRNA signature with potential diagnostic and prognostic relevance in RCC. In multivariable Cox regression analyses, higher *LINC01510* expression was associated with improved disease-free and overall survival, with hazard ratios below 1. *LINC01510* expression decreased with advancing disease stage and was associated with adverse clinicopathological features [35]. *LINC01510* expression has been reported to be approximately 49-fold higher in the normal renal cortex than in the reference tissues examined [38].

In the present study, we compared genome-wide DNAm profiles of localized and metastatic primary ccRCC and identified metastasis-associated differentially methylated regions (DMR) at the *LINC01354* and *LINC01510* loci.

We validated these findings using an independent pyrosequencing approach in primary RCC tissues, primary tumors from patients with distant metastases, and RCC metastatic tissues. These analyses revealed hypermethylation at several CpG sites and identified candidate loci for subsequent functional studies.

Our findings expand the spectrum of metastasis-associated epigenetic alterations in RCC and provide a basis for evaluating their potential prognostic and biological relevance.

## Material and methods

### Study design

To identify metastasis-associated candidate loci and DMR, we performed a discovery study using genome-wide DNAm profiling with Illumina EPIC arrays.

Candidate loci were subsequently validated by pyrosequencing, an independent quantitative method for measuring relative DNAm levels. Relative DNAm levels were determined in positive and negative controls, primary human cells, tumor cell lines, primary RCC tissues, and metastatic tissue samples, as previously described [18]. Relative methylation levels in paired tumor-adjacent normal tissues (adNs) and corresponding tumor tissues were analyzed using paired statistical comparisons. Independent tumor groups and metastatic tissue samples were evaluated using a cross-sectional study design.

### Study cohort

Pyrosequencing-based methylation analyses were performed in 145 primary RCC tissues, 124 paired adNs and 194 metastatic tissue samples obtained from 101 patients with metastatic RCC. Patient characteristics, metastatic tissue sampling, TNM classification, tumor grading, and tissue processing have been described previously [39–41] and are summarized in Supplemental Table S1. Ethical approval was granted by the ethical boards of Eberhard Karls University Tübingen and Hanover Medical School (No. 128/2003V and 1213–2011, approved 14 October 2011). Written informed consent was obtained from all patients. The study was conducted in accordance with the Declaration of Helsinki.

### Preparation of DNA and DNA methylation analysis

The histological estimation of tumor cell content, DNA isolation from frozen tissue sections and formalin-fixed paraffin-embedded (FFPE) tissue punches, and bisulfite conversion of DNA were performed as previously described [42, 43].

### DNA profiling

DNAm profiling was performed using Infinium Methylation EPIC array (Illumina, Inc.). DNA extracted from 22 primary RCCs without distant metastases (M0) and 22 primary RCCs with distant metastases (M+) was diluted to approximately 50 ng/µl concentration and then subjected to bisulfite conversion with the EZ DNA Methylation Kit (Zymo Research), following the supplier's alternative incubation conditions for the Illumina Infinium Methylation EPIC Array. The converted DNA samples were then hybridized to the EPIC array and scanned on an iScan instrument (Illumina, Inc.) according to the manufacturer's instructions (Document #1000000077299v0). The raw DNAm intensities were generated using the iScan control software (v2.3.0.0; Illumina, Inc.) and exported in IDAT format for further processing and analysis.

### Primary cells and tumor cell lines

Primary renal epithelial cells (RPTEC) and primary human prostate epithelial cells (PrEC) were purchased from Lonza (Basel, Switzerland). RCC cell lines (RCC-MF, RCC-HS, RCC-GS, ACHN, A498, 786-O, prostate cancer cell lines (PC-3, LNCaP, DU-145), urothelial carcinoma cell lines (T24, RT112, HB-CLS1, HB-CLS2, CLS-439, 5637), and the cervical cancer cell line HeLa were purchased from Cell Lines Services (Eppelheim, Germany). Cells were cultured according to the manufacturers’ protocol for five passages and were used solely for DNA extraction [44, 45].

### Pyrosequencing analysis

Pyrosequencing was used to analyze DNAm. PCR reactions and pyrosequencing template preparation have been described earlier [42, 46]. The *LINC01354* (PS_301) and *LINC01510* (PS_306, PS_307) pyrosequencing assays were designed by use of the PyroMark Assay Design 2.0 software (Qiagen, Hilden, Germany) and the hg19 genome assembly as provided by the UCSC table browser. Supplemental Table S2 contains the forward, reverse, and sequencing primer sequences, as well as the sequence to analyze for pyrosequencing. Genomic locations of CpG sites covered by pyrosequencing analysis are presented in Table 1.

**Table 1:** Comparison of candidate CpG sites obtained by DNA profiling and sites amenable to independent pyrosequencing analysis. Note that a total of four candidate CpG sites could be measured also in pyrosequencing analysis while other sites demonstrated a maximum distance of 20 bp to the next candidate CpG

Chr	Gen. start	Locus	DNA-profiling	Pyroseq. assay
chr1	234,667,497	LINC01354	–	PS_301-CG5
chr1	234,667,500	LINC01354	–	PS_301-CG4
chr1	234,667,503	LINC01354	–	PS_301-CG3
chr1	234,667,517	LINC01354	cg02550311	PS_301-CG2
chr1	234,667,525	LINC01354	cg07872560	PS_301-CG1
chr1	234,667,549	LINC01354	cg21546522	–
chr7	116,254,788	LINC01510	cg19196374	PS_306-CG2
chr7	116,254,825	LINC01510	cg10320618	PS_306-CG1
chr7	116,254,846	LINC01510	cg18821123	–
chr7	116,254,947	LINC01510	cg19102672	–
chr7	116,254,983	LINC01510	–	PS_307-CG1
chr7	116,255,184	LINC01510	cg03998317	–

### DNA methylation profiling and in silico bioinformatics analyses

DNAm profiling data were analyzed using the Bioconductor packages minfi and bumphunter in R [47–49]. After quality control, data were preprocessed and resulting *β*-values were used for further evaluation. DMR were identified using the bumphunter package. Candidate DMR were ranked according to their family-wise error rate (FWER) provided by the algorithm considering a permutation-based *p*-value adjusted for multiple testing and the spatial extension of the DMR identified.

In silico analyses of The Cancer Genome Atlas Kidney Renal Clear Cell Carcinoma Collection (TCGA-KIRC) data were performed using the R package TCGAbiolinks [50]. Data from the RNAInter database were incorporated [51], and pathway analyses were conducted using the ReactomePA R package [52]. Figures were generated using the R packages ggplot2 [53] and patchwork [54].

### Statistical analysis

All statistical analyses were conducted using R v4.3.0 software, RStudio®, and program libraries as specified before [55, 56].

Statistical analyses of pyrosequencing data were performed using CpG site-specific relative methylation values without data transformation. Tumor-specific hypermethylation in paired samples was assessed using two-sided paired *t*-tests, whereas independent group comparisons were performed using logistic regression models with age and sex as covariates. For patients with multiple metastatic tissue samples, methylation values were averaged before statistical analysis.

## Results

### Identification of candidate CpG sites by DNA profiling and pyrosequencing analysis

Genome-wide DNAm profiling of 22 M0 and 22 M+ primary ccRCCs identified ten candidate DMR with FWER values ≤ 0.03. Among these, two DMR were located within the *LINC01510* (rank 1; FWER < 0.001) and *LINC01354* (rank 4; FWER = 0.025) loci (Table 2).

**Table 2:** Ranking and characteristic of LINC-DMR identified by bumphunter analysis. Exclusively DMR affecting LINC gene loci (Rank, DMR, gen.Pos., Gene) and falling below the statistical cut off limit of fwer ≤ 0.05 (fwer), lengths of DMR (DMR length), number and specifications of named CpG sites (No. cg, cgName, Chr., Pos.) probed by Epic DNA methylation array analysis are shown. Note that additional CpG sites not considered or measurable by array analysis may be present within DMR

Rank	DMR, gen. Pos	Gene	DMR, len. (bp)	fwer	No. cg	cgName	Chr	Pos
1	chr7:116,254,788	LINC01510	396	0.000	5	cg19196374	chr7	116,254,788
						cg10320618	chr7	116,254,825
						cg18821123	chr7	116,254,846
						cg19102672	chr7	116,254,947
						cg03998317	chr7	116,255,184
4	chr1:234,667,517	LINC01354	32	0.025	3	cg02550311	chr1	234,667,517
						cg07872560	chr1	234,667,525
						cg21546522	chr1	234,667,549

As shown in Fig. 1, CpG sites within both candidate DMR exhibited significantly higher median DNAm in M+ primary RCC than in M0 tumors. The confidence intervals of the median methylation values did not overlap between M+ and M0 tumors, indicating significantly increased methylation in M+ primary RCC.

To validate the candidate loci using an independent method, three pyrosequencing assays (PS_301, PS_306, and PS_307; Table 1) were designed to interrogate either four candidate CpG sites or immediately adjacent CpG sites located within 20 bp (Table 2).

### Analysis of LINC01354 and LINC01510 associated CpG methylation in cell line models

**Fig. 2:**
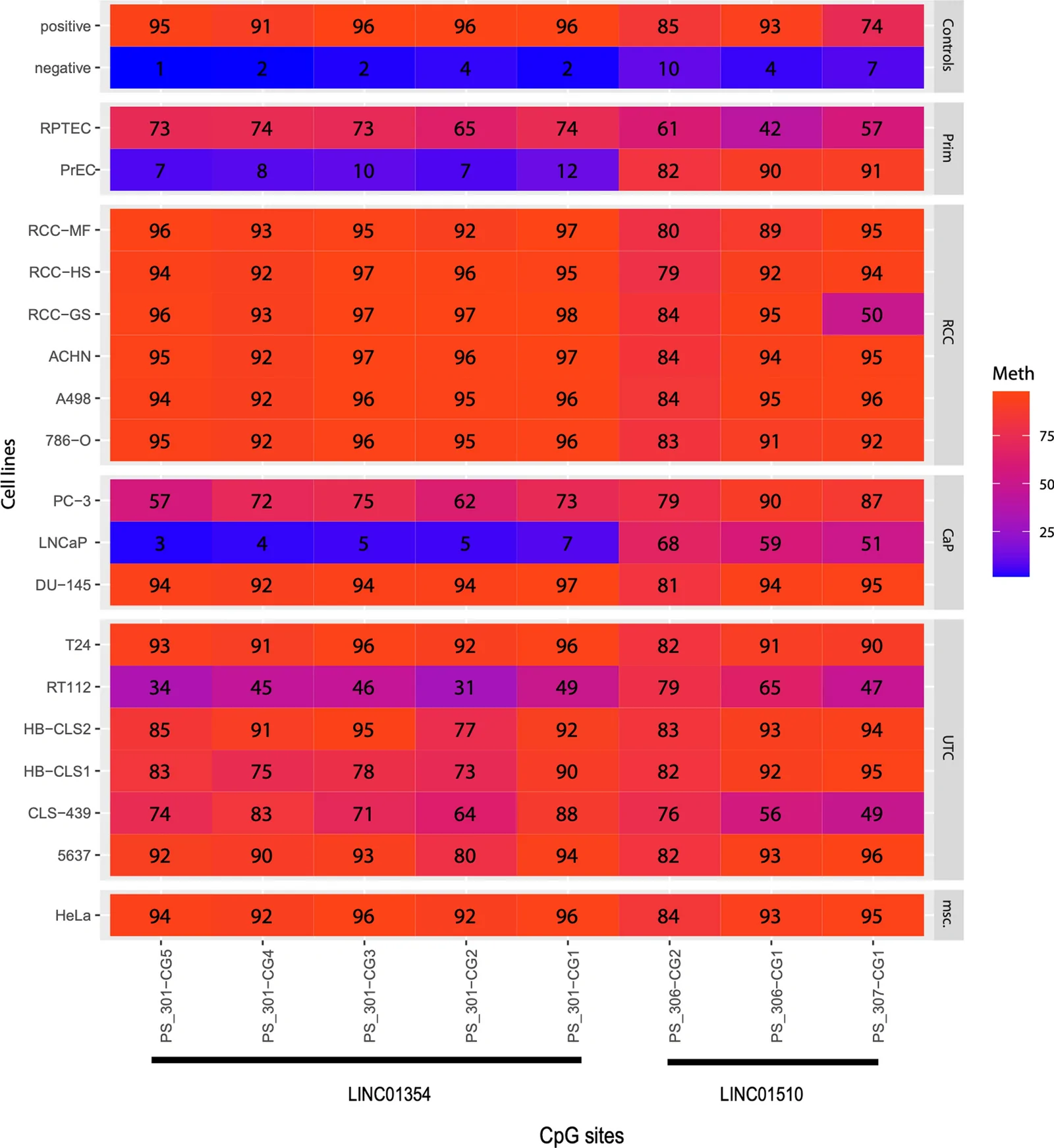
Heatmap illustration of relative DNA methylation levels (percentage) of candidate CpG sites following pyrosequencing in artificial positive and negative DNA controls, primary renal cells (Prim), renal cell carcinoma (RCC), prostate cancer (CaP), urothelial cancer (UTC), and additional cancer cell line models (msc). Methylation levels are visualized using a color gradient from zero (blue) to 100% methylation (red) and given also as numeric values. CpG sites are arranged according to their genomic order

DNAm of the candidate DMR was first analyzed by pyrosequencing in 18 cell models, including RPTEC, PrEC, six RCC cell lines, three prostate cancer cell lines, six urothelial carcinoma cell lines, and one cervical cancer cell line. RCC cell and cervical cancer cells showed generally high methylation levels (50–98%), whereas prostate and urothelial cancer cell lines displayed greater variability (Fig. 2). The *LINC01354* locus showed particularly lower methylation levels in prostate cancer cells, representing a less aggressive prostate cancer phenotype, also showing reduced medium methylation for the *LINC01510* (PS_306)-associated sites. Methylation levels mostly exceeding 90% were observed for both candidate regions in the more aggressive DU-145 cell line. Cervical cancer cells showed high relative methylation between 84 and 96%.

### Hypermethylation analysis of LINC01354 and LINC01510 in tumor-adjacent normal tissue and primary tumor tissue

**Fig. 3:**
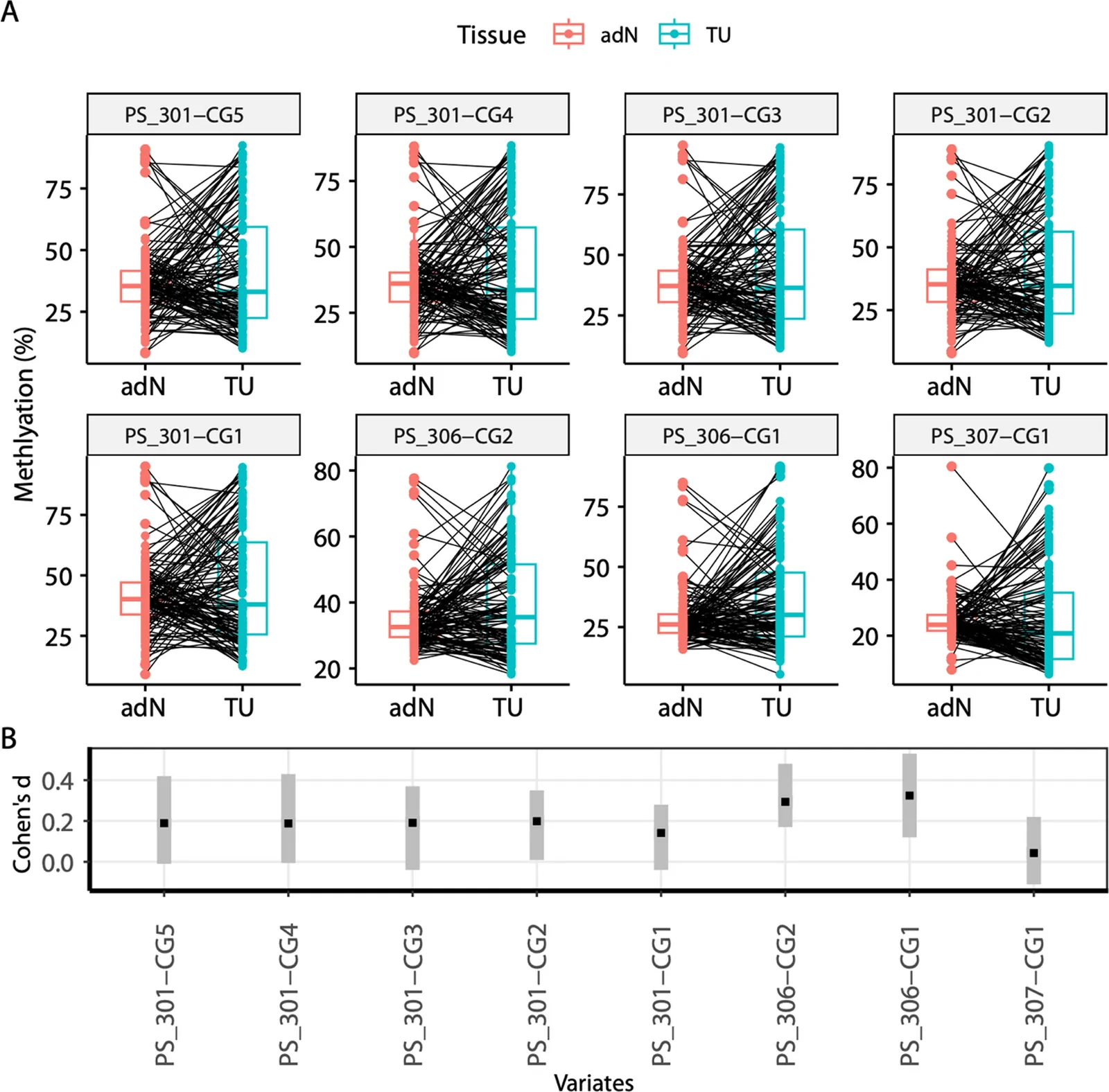
**A** Paired strip plots illustrating CpG methylation levels in pairs of tumor-adjacent normal (adN, apricot) and primary tumor (TU, turquoise) tissues for selected loci within the LINC01354 (PS_301) and LINC01510 (PS_306 and PS_307) genes, assessed by pyrosequencing. Each pair is connected by a black line, representing matched samples from the same patient. Methylation levels are shown as percentages. **B** Forest plot presentation of statistical effect size of methylation differences between paired samples using Cohen’s d. Values are displayed as black squares with corresponding confidence intervals (gray bars) for all CpG sites in genomic order. Corresponding statistical results are provided in Table 3

For comparison of paired tissues, 124 pairs of adNs and corresponding tumor tissues were analyzed by pyrosequencing. Two out of eight analyzed CpG sites (PS_306_CG1 and PS_306_CG2—both located within *LINC01510*) showed significant hypermethylation with a small effect size according to Cohen’s *d* (Table 3). Inspecting the paired strip plots all *LINC01354* CpG sites (PS_301) exhibited heterogeneity as substantial subsets of tissue pairs showed either moderate hypomethylation or a more pronounced hypermethylation effect in tumors (Fig. 3). A similar situation was found for *LINC01510* (PS_306, PS_307) although the group of tissues showing hypermethylation appeared to be smaller in comparison to *LINC01354* data.

**Table 3:** Comparison of CpG methylation levels in paired tumor-adjacent normal and primary RCC samples for loci within the LINC01354 (PS_301) and LINC01510 (PS_306 and PS_307) genes, as obtained by pyrosequencing. Two-sided paired t-tests were performed to evaluate group differences between paired samples. Benjamini–Hochberg adjusted *p*-values (*p*.adj) and corresponding significance levels (*p < 0.05; **p< 0.005; ns, not significant) are shown alongside effect sizes (Cohen’s d) and their magnitude classification

Assay	*p*.adj	*p*.adj.signif	Cohens_d	Magnitude
PS_301-CG5	0.184	ns	0.19	Negligible
PS_301-CG4	0.184	ns	0.19	Negligible
PS_301-CG3	0.184	ns	0.19	Negligible
PS_301-CG2	0.180	ns	0.20	Negligible
PS_301-CG1	0.240	ns	0.14	Negligible
PS_306-CG2	0.010	*	0.29	Small
PS_306-CG1	0.004	**	0.32	Small
PS_307-CG1	0.638	ns	0.04	Negligible

### Hypermethylation of *LINC01354* and *LINC01510* in aggressive primary cancers

**Fig. 4:**
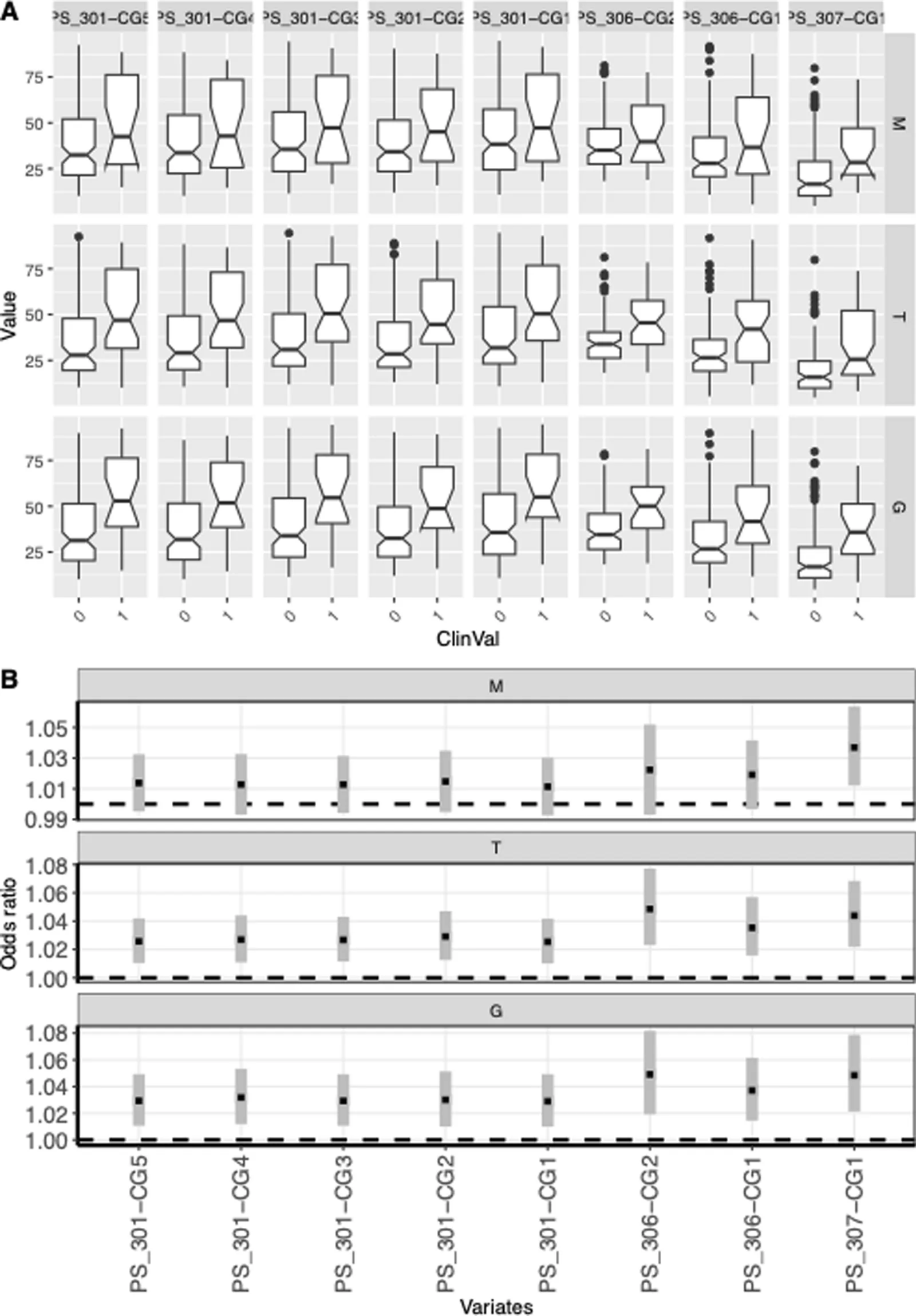
**A** Boxplot analysis of CpG methylation levels in primary RCC tissues stratified by metastatic status (M0 vs. M1), tumor stage (low < T3 vs. high ≥ T3), and histological grade (low < G3 vs. high ≥ G3) for selected CpG sites. Boxes represent the interquartile range (25th–75th percentile) with median values indicated; notches denote the confidence interval around the median, whiskers extend to 1.5× the interquartile range, and outliers are shown as black dots. **B** Forest plot showing CpG site-specific odds ratios (ORs) and corresponding confidence intervals derived from bivariate logistic regression analyses for the group comparisons shown in (**A**). CpG sites are presented in genomic order. Corresponding statistical results are provided in Supplementary Table S3

To analyze whether methylation of the candidate regions of *LINC01354* and *LINC01510* show association with clinical parameters including the state of distant metastasis, low- and high-stage tumors, and low- and high-grade histopathological differentiation of tumor cells we carried out tumor group comparisons using logistic regression following if applicable dichotomization of the explanatory variables. Only PS_307-CG1 showed significantly higher methylation in M+ tumors than in M0 tumors (OR 1.04 per 1% increase in methylation; *p* = 0.031). However, visual inspection of the boxplots for methylation distribution in M0 and M+ tumors also indicated the presence of high methylation values for the other CpG sites measured in M+ tumors (Fig. 4, Supplemental Table 3A) and resulting in similar odds ratios. The lack of statistical significance for the remaining loci is likely attributable to the limited number of M+ cases and the high variability in methylation levels, which resulted in wider confidence intervals and reduced statistical power. In contrast, the comparison of low- and high-stage tumors exhibited significant differences in means exhibiting hypermethylation in high-stage tumors for all the CpG sites (OR 1.03–1.05, all *p* = 0.001 following Benjamini–Hochberg adjustment for multiple testing) in both candidate regions (Fig. 4, Supplemental Table 3B). Similarly, high-grade tumors were also found to be associated with higher methylation of all loci measured (OR 1.03–1.05, all *p* = 0.003) (Fig. 4, Supplemental Table 3C). The consistently highest values, in relation to the other candidate sites, for the odds ratios in tumor group comparisons of state of distant metastasis, high-stage and high-grade tumors were found for the PS_306-CG2 and PS_307-CG2 and -CG1, all located in *LINC01510/COMETT*. The covariates age and sex did not demonstrate a significant effect in any of the tumor group comparisons (data not shown).

### Metastatic tissue-specific hypermethylation of *LINC01354* and *LINC01510*

**Fig. 5:**
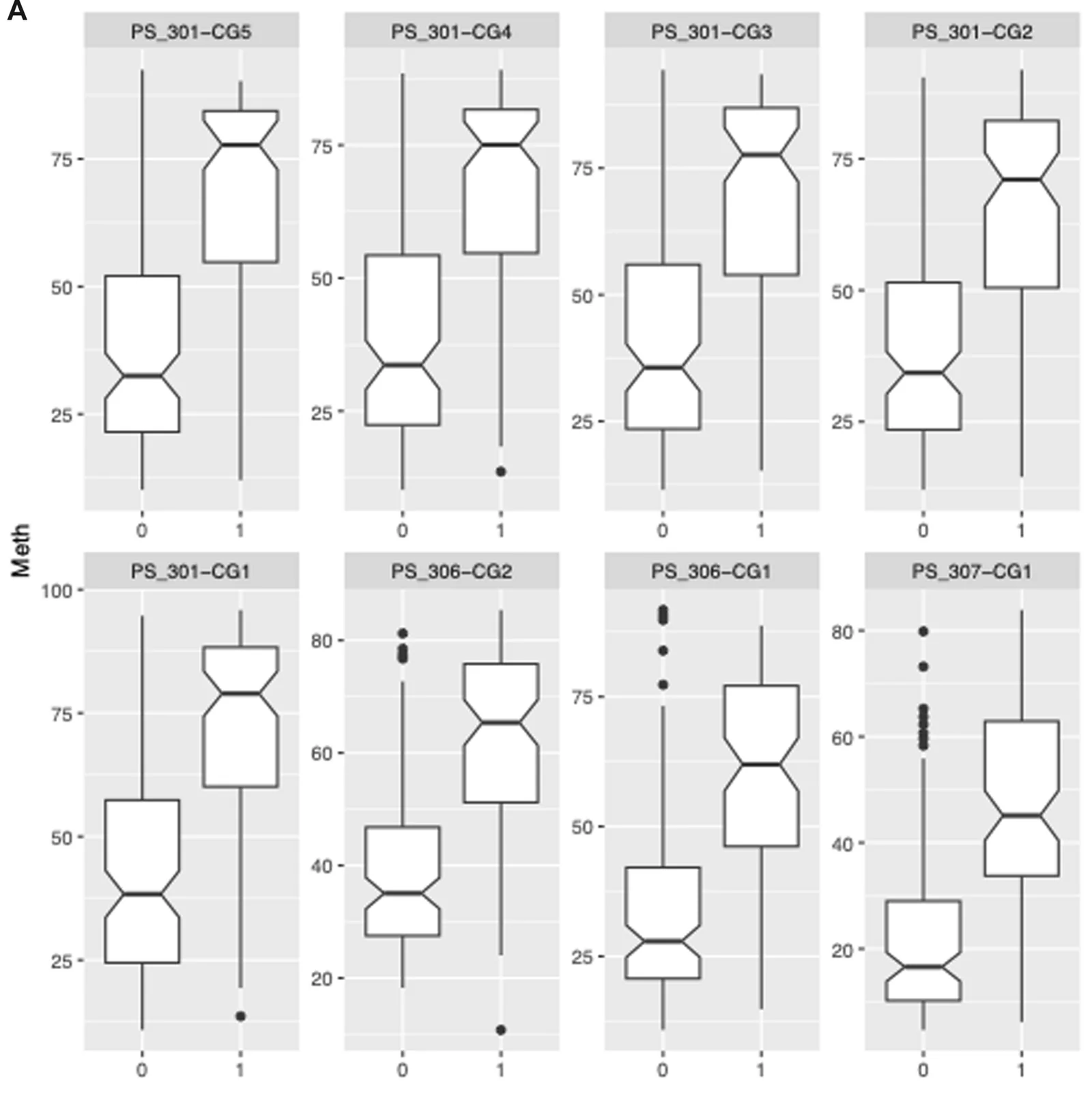

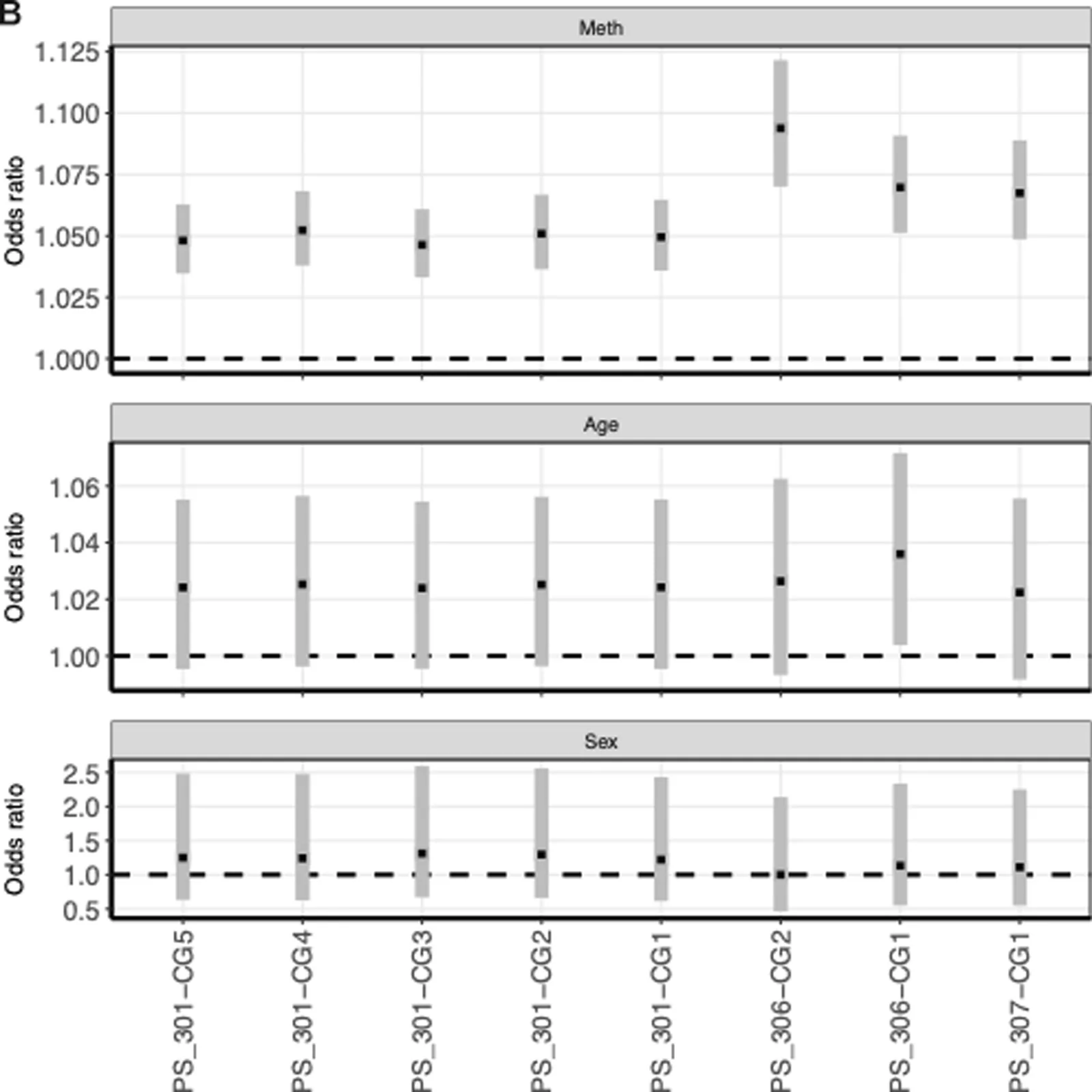
**A** Boxplot analysis of CpG methylation levels in primary non-metastatic RCC tissues (0) and metastatic tissue samples (1) for selected CpG sites. Boxplot elements are displayed as described in Fig. 4. **B** Forest plot showing CpG site-specific odds ratios (ORs) with corresponding confidence intervals following logistic regression analyses for the categorical response variables of metastatic state of non-metastatic primary tissues and metastatic tissue samples and the predictor variables CpG methylation, age, and sex

The analysis of metastatic tissues obtained from RCC patients in comparison to primary localized RCC tissues revealed substantially higher methylation of all candidate loci of *LINC01354* as well as *LINC01510* in RCC metastases. Confidence intervals of the median DNAm values, as indicated by the notches around the medians in boxplots, showed no overlap (Fig. 5A). Statistical analysis demonstrated odds ratios of 1.05–1.09 per one percent change in methylation (all *p*-values < 0.001, Table 4). Mean group methylation values differed by approximately 25–30 percentage points (PS_301-CG1, PS_306-CG2) corresponding to odds ratios of up to 8.9 (PS_306-CG2) when referred to group mean differences. Forest plot analysis of CpG-specific odds ratios and confidence intervals also showed that hypermethylation effects in metastases were most pronounced for the *LINC01510* loci (Fig. 5B, row “Meth”, PS_306-CG2, -CG-1 and PS_307-CG1 sites). Except for PS_306-CG1, for which age showed a potential effect on the odds of metastatic tissue, none of the other candidate CpG sites were associated with the covariates age or sex (Fig. 5B, rows “Age” and “Sex”).

**Table 4:** Association between CpG methylation levels and metastatic state of RCC tissue comparing primary localized RCC tissue and metastatic tissue samples by use of logistic regression considering age and sex as covariates. Odds ratios (ORs) refer to 1% increase in methylation for individual CpG sites within the LINC01354 (PS_301) and LINC01510 (PS_306 and PS_307) loci. Metastatic state was used as the outcome variable. Confidence intervals (conf.low, conf.high) and Benjamini–Hochberg adjusted *p*-values (*p*.value.adj) are shown. Age and sex did not significantly contribute to the statistical models (data not shown)

Var	OR	conf.low	conf.high	*p*.adj	sig.adj
PS_301-CG5	1.048	1.035	1.063	< 0.001	***
PS_301-CG4	1.052	1.038	1.068	< 0.001	***
PS_301-CG3	1.046	1.033	1.061	< 0.001	***
PS_301-CG2	1.051	1.036	1.067	< 0.001	***
PS_301-CG1	1.050	1.036	1.065	< 0.001	***
PS_306-CG2	1.094	1.070	1.122	< 0.001	***
PS_306-CG1	1.070	1.051	1.091	< 0.001	***
PS_307-CG1	1.067	1.049	1.089	< 0.001	***

### In silico analysis of *LINC01354* associated CpG sites measured in the TCGA-KIRC project

Comparison with the TCGA-KIRC project identified one shared CpG site (cg21546522). The corresponding in silico analysis of TCGA-KIRC cg21546522 data as well as of the adjacent cg19856705, and cg10811426 sites, located 5’ within 100 bp (Supplemental Table 4), demonstrated an association between hypermethylation and almost all adverse clinical and disease outcome parameters beside lymph node metastasis. Significant relationships were detected for the state of distant metastasis, high-stage and high-grade ccRCC (Supplemental Table 5). A highly significant association with overall survival was observed in univariate analyses for all three CpG sites and in multivariate Cox regression analyses for cg19856705 and cg21546522 (Supplemental Table 6).

### In silico analysis of *LINC01354* DNAm and mRNA expression correlation

Analysis of the TCGA-KIRC project showed that assessment of potential epigenetic silencing was only possible for *LINC01354*, as both DNAm and gene expression data were available for the cg10811426, cg19856705, and cg21546522 CpG sites. In silico analysis revealed that methylation of these CpG sites showed significant negative correlations with mRNA expression of *LINC01354*, both in adNs as well as in the tumor tissue group (Supplemental Fig. 2, Supplemental Table S7). The correlation coefficients ranged between R = − 0.41 to − 0.51 for tumor tissues and − 0.50 to − 0.59 in adNs (*p* < 1*10^–8^ in tumor tissues, *p* < 0.014 in adNs, Benjamini–Hochberg adjusted). Intra-CpG site comparison of methylation in all tissues showed correlation coefficients of 0.90–0.96.

### In silico analysis of *LINC01354* and *LINC01510* regulatory features

**Fig. 6:**
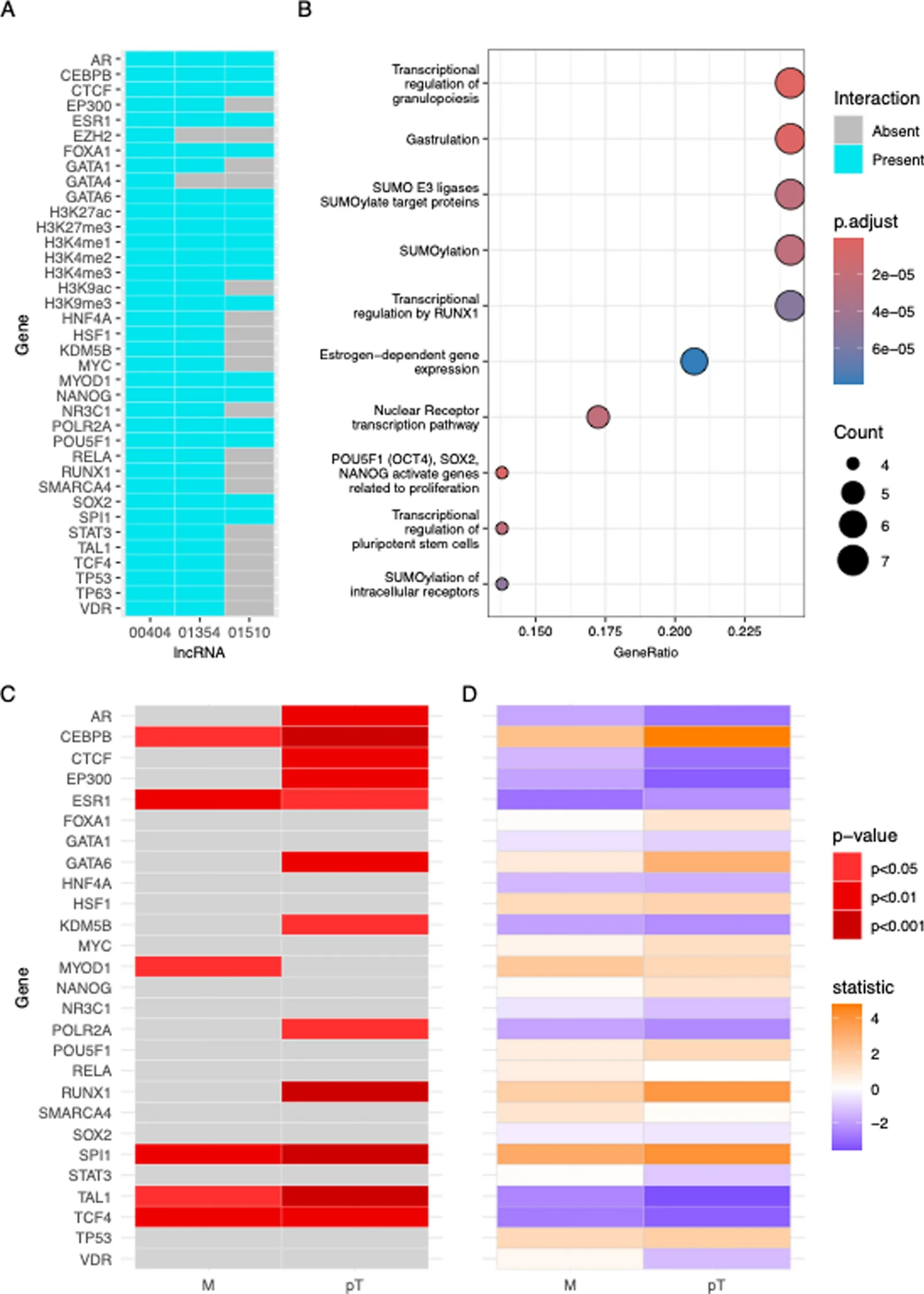
In silico analysis of the RNAinter and TCGA-KIRC database. **A** Interaction heatmap of protein-lncRNA interactions as obtained by ChIP analysis and reported by the RNAINter database. Note that LINC00404, − 01354, and − 01510 share all of the reported target molecules as indicated by light-blue colored heatmap tiles. **B** Target protein enrichment analysis illustrating the relative proportion of identified lncRNA target genes attributed to specific signal pathways as specified on the left y-axis. Moreover, the absolute number (circle diameter) of target molecules and estimated significance of signal pathway assignment (color gradient) is shown. **C**, **D** Significance and direction of mRNA expression alterations evaluated for the lncRNA target genes using TCGA-KIRC data for high and low stage tumor groups (pT) as well as the metastatic state of tissues (M) using age-adjusted logistic regression. *P*-values < 0.05 were indicated by red tiles (**C**) while hyper- and hypoexpression is indicated by orange and purple color coding, respectively referring to the test statistics (**D**)

We also asked the RNAinter database [51] for measured interactions of *LINC01354* and *LINC01510*, supplemented by *LINC00404* as a metastasis-relevant RNA recently reported by our group [19], with histones, transcription factors, and RNA. We found that three lncRNAs share a large part of interactions with proteins and epigenetically modified histones (Fig. 6A). All the *LINC01354* and *LINC00404* targets matched and all the reported significant interaction partners of *LINC01510* were also subset of the *LINC01354* and *LINC00404* interacting molecules. Pathway enrichment analysis of proteins of specific signaling pathways revealed that genes involved in gastrulation, regulation of granulopoiesis, SUMOylation, and RUNX1 transcriptional regulation were significantly enriched (Fig. 6B). Considering that expression data of most of these candidate lncRNA target proteins were evaluated before by the TCGA-KIRC project we eventually carried out a corresponding in silico analysis to clarify whether expression alterations of lncRNA interacting transcription factors were associated with an adverse clinical status of ccRCC. We found that a substantial part of the putative target proteins showed significant alterations in aggressive, i.e. high-stage or metastatic RCC, and, that alterations to a substantial part is characterized by loss of expression in those tumor subsets (Fig. 6C, D).

## Discussion

Identifying molecular alterations that contribute to metastatic progression is essential for improving the diagnosis, prognostication, and treatment of RCC. Increasing evidence suggests that dysregulation of non-coding RNAs contributes to tumor progression in ccRCC.

LncRNAs, microRNAs, and pseudogene-derived transcripts are frequently affected by epigenetic alterations, including changes in DNA methylation, that have been associated with aggressive tumor behavior and poor clinical outcome.

In line with these observations, previous work from our group and others demonstrated that epigenetic deregulation of non-coding RNA genes can be linked to tumor progression and metastasis in RCC.

We recently reported that DNAm of *miR124-3*, representing oncogenic microRNAs, and *LINC00404*, representing lncRNAs, is associated with distant metastasis and that both loci are hypermethylated in metastatic RCC tissues [19, 43]. Together, these findings indicate that altered DNAm of regulatory RNA loci is recurrently associated with RCC progression and metastatic disease [57]. Whereas *LINC00404* and *miR124-3* were identified through in silico candidate searches using the TCGA-KIRC data, DMR located within *LINC01354* and *LINC01510* were identified in a de novo search using DNAm profiling and the improved successor DNAm profiling arrays enabling the analysis of additional CpG sites not covered by the HM450K array used in the TCGA-KIRC project. Seven of the eight candidate CpG sites identified in the present study were not covered by the HM450K array. Although cg21546522 was included in the HM450K array, differential methylation could not be evaluated because the site showed little to no variability in the TCGA-KIRC project (data not shown).

Both DMR ranked among the top candidates for metastasis-associated methylation changes and showed significantly increased methylation in primary M+ RCC (Table 1). Inspection of *β*-values obtained from methylation profiling suggested that for *LINC01354* DNAm gains in M+ tumors were not uniformly but rather showed a bimodal distribution, indicating substantial intertumoral heterogeneity (Fig. 1).

Analysis of RCC, urothelial, prostate, and cervical cancer cell lines revealed generally high methylation levels, indicating a possible relevance of these alterations across a broad range of human cancers. Although not all analyzed cell lines showed very high methylation levels, an overall trend toward high methylation was observed across the different cancer cell types. Individual cell lines, such as the urothelial cancer cell lines RT112 and CLS-439 and the prostate cancer cell lines PC-3 and LNCaP, showed relatively lower mean methylation levels at either one or both loci, potentially reflecting biological heterogeneity among the analyzed cancer cell lines (Fig. 2).

Comparison of paired adN and tumor tissues revealed considerable heterogeneity in methylation patterns. Whereas some tumors showed hypermethylation, others exhibited hypomethylation, resulting in limited overall effect sizes and a lack of statistical significance for several *LINC01354* loci (Fig. 3; Table 3).

Comparisons of independent tumor groups demonstrated significant hypermethylation in high-stage and high-grade tumors across all measured CpG sites. In contrast, the association with metastatic status was less pronounced, with a significant association observed only for the PS_307-CG1 site in *LINC01510* (Fig. 4; Supplemental Table 3A). Although several other CpG sites showed odds ratios indicating a tendency toward higher methylation in M+ tumors, these associations did not reach statistical significance. The imbalance between the M0 and M+ group sizes and substantial intertumoral heterogeneity may have reduced statistical power. Larger cohorts are required to determine whether methylation at these loci is associated with M+ status.

We additionally compared metastatic tissue samples with primary tumors from M0 patients in a more balanced study design. Notably, this analysis revealed substantially higher odds ratios and highly significant *p*-values for all investigated CpG sites, accompanied by a narrower distribution of methylation values (Fig. 5A, B; Table 4). These findings demonstrate that hypermethylation at both loci is more pronounced in established metastatic tissue than in localized primary tumors, indicating an association with the metastatic phenotype.

The CpG sites within *LINC01510* demonstrated the most pronounced metastasis-associated hypermethylation effects among the RNA regulatory loci analyzed in our previous and present studies, including *LINC00404*, *LINC01354* and *LINC01510.*

To validate our findings using an independent dataset, we analyzed TCGA-KIRC data for three *LINC01354*-associated CpG sites: cg21546522, the only CpG site shared with our methylation analysis, and the two adjacent CpG sites cg19856705 and cg10811426, which were available only in the TCGA-KIRC dataset. We found significant associations between increased methylation and high-stage, high-grade, and M+ tumors, supporting our findings and suggesting that the metastasis-associated *LINC01354* DMR extends beyond the region covered by our methylation analysis toward the 5′ direction. In silico analysis of TCGA-KIRC data for overall survival analysis revealed univariable and multivariable significance for the cg21546522 and cg19856705 sites, further supporting an association between increased methylation at these sites and aggressive RCC characteristics.

TCGA-KIRC analyses further demonstrated a significant negative correlation of DNAm in these sites and *LINC01354* mRNA expression. Although this finding is consistent with a potential epigenetic regulatory relationship, the modest correlation coefficients and the observational character of the analysis do not establish methylation-mediated silencing.

The potential relevance of *LINC01354* is further supported by the finding that, in addition to DNAm, mRNA expression alterations were also associated with aggressive RCC characteristics. *LINC01354* mRNA expression was significantly reduced in high-stage tumors as well as in tumors from patients with presence of lymph node or distant metastases.

Results for *LINC01510* were less consistent. Apart from the problem that appropriate CpG sites in or adjacent to the gene were not available in the TCGA-KIRC analysis, expression of mRNA, though similar in trend, failed to demonstrate statistical significance when correlated with adverse clinical parameters. Inspection of expression values obtained for paired adN and tumor samples revealed a considerable degree of heterogeneity displaying distinguishable subgroups showing either distinctive hypo- or hyperexpression in tumor tissues. This heterogeneity may have limited the ability of group-based statistical comparisons.

Alternatively, the greater variability in *LINC01510* expression may indicate more complex regulatory mechanisms, as regulatory RNAs often interact with multiple target molecules, including other RNAs and transcription factors. To investigate this possibility, we queried the RNAInter database [51]. We found that all *LINC01510* targets were also part of the *LINC01354* target structures, and these also showed substantial overlap with the target molecules of *LINC00404*, recently identified by our group to show strong association of methylation and expression with the metastatic state of renal tissues.

The interaction networks of *LINC01354* and *LINC01510* included several transcriptional regulators. Pathway enrichment analyses identified processes involved in gastrulation, granulopoiesis, transcriptional regulation of pluripotent stem cells, RUNX1 signaling, SUMOylation, and PD-L1 regulation. Several mechanisms have been implicated in RCC biology [58–71]. These analyses are exploratory and may provide hypotheses regarding potential regulatory contexts of *LINC01354* and *LINC01510* for subsequent functional studies and experimental validation.

In conclusion, our findings extend current evidence of epigenetic alterations of lncRNA loci in ccRCC by demonstrating tumor- and metastasis-associated hypermethylation of *LINC01354* and *LINC01510*.

The pronounced metastasis-associated hypermethylation of *LINC01354* and *LINC01510* observed in metastatic tissue supports an association with established metastatic disease. However, the less consistent differences between primary M0 and M+ tumors do not provide predictive values for metastatic risk.

Furthermore, our findings contribute to the characterization of ccRCC within the framework of altered DNAm signatures defining molecular subtypes, prognosis, and tumor aggressiveness, thereby offering potential utility for clinical risk stratification. A major limitation of the present study is its observational design. The statistical relationships do not allow causal relationships between DNAm, lncRNAs expression, and disease progression. Functional studies are required to determine the biological impact of DNAm at the *LINC01354* and *LINC01510* loci.

Future studies should therefore confirm functional relevance and clarify whether DNAm of *LINC01354* and *LINC01510* can improve existing prognostic models and patient outcome prediction [18, 72–75].

## Conclusion

Here, we identified *LINC01354* and *LINC01510* as metastasis-associated epigenetically deregulated lncRNA loci in ccRCC. DNAm profiling revealed metastasis-associated hypermethylation, particularly pronounced in established metastatic lesions. Evaluation of independent TCGA-KIRC data supported an association between increased CpG methylation at the *LINC01354* locus, reduced *LINC01354* expression, and aggressive clinicopathological features. Further studies are necessary to determine the functional relevance and potential prognostic value of these methylation changes.

## Supplementary Information

Additional file 1 (DOCX 3163 kb)

Additional file 2 (DOCX 27 kb)

## Data Availability

All additional data not included in the manuscript are available from the corresponding author upon reasonable request.

## Abbreviations

**Abstract**
adN: Tumor-adjacent normal tissue
ccRCC: Clear cell renal cell carcinoma
ceRNA: Competing endogenous RNA
COMETT: Cytosolic oncogenic mRNA antisense to MET transcript
DMR: Differentially methylated regions
DNAm: DNA methylation
FFPE: Formalin-fixed paraffin-embedded
FWER: Family-wise error rate
HIF-2α: Hypoxia-inducible factor 2 alpha
lncRNA: Long non-coding RNA
M0: Without distant metastases
M+: With distant metastases
mRCC: Metastatic RCC
NSCLC: Non-small cell lung cancer
PrEC: Primary human prostate epithelial cells
RCC: Renal cell carcinoma
RMAMS: Renal metastasis-associated methylation signature
RPEC: Primary renal epithelial cells
TCGA: The Cancer Genome Atlas
TCGA-KIRC: The Cancer Genome Atlas Kidney Renal Clear Cell Carcinoma Collection
VHL: Von Hippel-Lindau
