# Supplementary material for: Metastasis-associated DNA methylation of the lncRNAs LINC01354 and LINC01510 in clear cell renal cell carcinoma
Source: Clin Epigenetics. 2026 Oct 13;18:185. doi: 10.1186/s13148-026-02252-4 (PMC13663206; doi:10.1186/s13148-026-02252-4)
Supplement: Supplementary file 1 [file 13148_2026_2252_MOESM1_ESM.docx]

# Supplemental Figure 1

Genomic context of the LINC01510 gene, including the location of CpG islands, the approximate positions of candidate CpG sites, and DNA methylation patterns at selected CpG sites in cancer cell line models, as reported by the ENCODE project.

# Supplemental Figure 2

Correlation analysis of *LINC01354* DNA methylation and mRNA expression using TCGA-KIRC data. *β*-values were correlated with mRNA expression data for 24 adN (A) and 294 tumor tissue samples (B) as well as methylation values of neighbored CpG sites. The corresponding correlation coefficients and *p*-values are presented in Supplemental Table 7.

Correlation analysis of *LINC01354* DNA methylation and mRNA expression using the TCGA-KIRC dataset. DNA methylation (*β*-values) was correlated with mRNA expression levels in 24 tumor-adjacent normal tissue (adN) samples (A) and 294 tumor tissue samples (B), as well as with DNA methylation levels of neighboring CpG sites. The corresponding correlation coefficients and *p*-values are provided in Supplementary Table S7.
