# Supplementary material for: Metastasis-associated DNA methylation of the lncRNAs LINC01354 and LINC01510 in clear cell renal cell carcinoma
Source: Clin Epigenetics. 2026 Oct 13;18:185. doi: 10.1186/s13148-026-02252-4 (PMC13663206; doi:10.1186/s13148-026-02252-4)
Supplement: Supplementary file 2 [file 13148_2026_2252_MOESM2_ESM.docx]

# Supplemental Table S1

|  | Paired tissue cohort | Primary RCC cohort | Localized RCC cohort (M0) | Metastatic RCC cohort (M+) |
| --- | --- | --- | --- | --- |
|  |  |  |  |  |
| Total | 124 (100%) | 145 (100%) | 121 (100%) | 101 (100%) |
| Sex |  |  |  |  |
| M | 76 (61%) | 89 (61%) | 73 (60%) | 71 (70%) |
| F | 48 (39%) | 56 (39%) | 48 (40%) | 30 (30%) |
| Age, median (range) | 65 (54, 70) | 65 (54, 70) | 65 (54, 71) | 66 (59, 73) |
|  |  |  |  |  |
| Histological subtype |  |  |  |  |
| ccRCC (%) | 124 (100%) | 145 (100%) | 121 (100%) | 101 (100%) |
|  |  |  |  |  |
| Histological grade |  |  |  |  |
| Low grade |  | 122 (84%) |  |  |
| High grade |  | 23 (16%) |  |  |
| Tumor stage |  |  |  |  |
| Low stage |  | 96 (66%) |  |  |
| High stage |  | 49 (34%) |  |  |
| Metastatic status |  |  |  |  |
| M0 |  | 121 (83%) |  |  |
| M+ |  | 24 (17%) |  |  |
|  |  |  |  |  |

Clinicopathological characteristics of the paired tissue cohort, primary RCC cohort, localized RCC cohort (M0), and metastatic RCC cohort (M+). Data are presented as n (%) or median (range). RCC, renal cell carcinoma; ccRCC, clear cell renal cell carcinoma; M0, patients without distant metastasis; M+, patients with distant metastasis; low grade, <G3; high grade, ≥G3; low stage, <pT3; high stage, ≥pT3.

# Supplemental Table S2

| Assay | Type | Seq. |
| --- | --- | --- |
| PS_301 | FW | AGTTATGATGAGGGTTAGGAGGT |
|  | RV | AAATCTCCAACCCTACTCAAACACC |
|  | SQ | CCTACTCAAACACCACATA |
|  | StoA | CRCAAAACCRACTACATTCCCACRACRACRACCTCCTAACCC |
| PS_306 | FW | TTTTTTGTTTTTTGTTTAAAGGTTGTAGG |
|  | RV | ACACAAAAAAAAAATCCAAATTAACAAATACTCT |
|  | SQ | AATCCAAATTAACAAATACTCTCTC |
|  | StoA | RATTACTCCCTAATTACAATTAATAAACAACCTATTCRCCTACAACCTTTAAACAAAAAACAA |
| PS_307 | FW | ATATGATGAAGGTAGAGTTAAAGATTTTAGTTT |
|  | RV | CAAATATTTCCTCCTTAAATAAACCATCTA |
|  | SQ | TTAGTTTATTAATAATTTGGAAAG |
|  | StoA | AYGTTAATTTTGGATTTAGTAAATATA |

Oligonucleotide sequences used for pyrosequencing analysis of CpG methylation at the LINC01354 (PS_301) and LINC01510 (PS_306 and PS_307) loci. Primer types include forward (FW), reverse (RV), sequencing (SQ), and sequence to analyze (StoA). All nucleotide sequences are provided in the 5′-3′ orientation. FW, forward primer; RV, reverse primer; SQ, sequencing primer; StoA, sequence to analyze; PS, pyrosequencing assay.

# Supplemental Table S3A

| Var | OR | Lower CI | Upper CI | *p*.adj |
| --- | --- | --- | --- | --- |
| PS_301-CG5 | 1.01 | 1.00 | 1.03 | 0.226 |
| PS_301-CG4 | 1.01 | 0.99 | 1.03 | 0.226 |
| PS_301-CG3 | 1.01 | 0.99 | 1.03 | 0.226 |
| PS_301-CG2 | 1.01 | 0.99 | 1.03 | 0.226 |
| PS_301-CG1 | 1.01 | 0.99 | 1.03 | 0.232 |
| PS_306-CG2 | 1.02 | 0.99 | 1.05 | 0.226 |
| PS_306-CG1 | 1.02 | 1.00 | 1.04 | 0.226 |
| PS_307-CG1 | 1.04 | 1.01 | 1.06 | 0.031 |

Association between CpG methylation levels and metastatic status determined by multivariable logistic regression analysis comparing localized primary RCC tumors (M0) and primary RCC tumors with distant metastasis (M+), adjusted for age and sex. Odds ratios (ORs) are given per 1% increase in DNA methylation for individual CpG sites within LINC01354 (PS_301) and LINC01510 (PS_306 and PS_307). Metastatic status (M0 vs. M+) served as the binary outcome variable. Corresponding 95% confidence intervals and Benjamini-Hochberg-adjusted p-values are shown. Age and sex were not significantly associated with metastatic status (data not shown). RCC, renal cell carcinoma; PS, pyrosequencing assay; CG, CpG site; OR, odds ratio; CI, confidence interval; *p*.adj, Benjamini-Hochberg-adjusted *p*-value; M0, patients without distant metastasis; M+, patients with distant metastasis.

# Supplemental Table S3B

| Var | OR | Lower CI | Upper CI | *p*.adj |
| --- | --- | --- | --- | --- |
| PS_301-CG5 | 1.03 | 1.01 | 1.04 | 0.001 |
| PS_301-CG4 | 1.03 | 1.01 | 1.04 | 0.001 |
| PS_301-CG3 | 1.03 | 1.01 | 1.04 | 0.001 |
| PS_301-CG2 | 1.03 | 1.01 | 1.05 | 0.001 |
| PS_301-CG1 | 1.03 | 1.01 | 1.04 | 0.001 |
| PS_306-CG2 | 1.05 | 1.02 | 1.08 | 0.001 |
| PS_306-CG1 | 1.04 | 1.02 | 1.06 | 0.001 |
| PS_307-CG1 | 1.04 | 1.02 | 1.07 | 0.001 |

Association between CpG methylation levels and tumor stage determined by multivariable logistic regression analysis comparing low-stage (<pT3) and high-stage (≥pT3) RCC tumors, adjusted for age and sex. Odds ratios (ORs) are given per 1% increase in DNA methylation for individual CpG sites within LINC01354 (PS_301) and LINC01510 (PS_306 and PS_307). Tumor stage (low stage vs. high stage) served as the binary outcome variable. Corresponding 95% confidence intervals and Benjamini-Hochberg-adjusted p-values are shown. Age and sex were not significantly associated with tumor stage (data not shown). RCC, renal cell carcinoma; PS, pyrosequencing assay; CG, CpG site; OR, odds ratio; CI, confidence interval; *p*.adj, Benjamini-Hochberg-adjusted p-value; low stage, <pT3; high stage, ≥pT3.

# Supplemental Table S3C

| Var | OR | Lower CI | Upper CI | *p*.adj |
| --- | --- | --- | --- | --- |
| PS_301-CG5 | 1.03 | 1.01 | 1.05 | 0.003 |
| PS_301-CG4 | 1.03 | 1.01 | 1.05 | 0.003 |
| PS_301-CG3 | 1.03 | 1.01 | 1.05 | 0.003 |
| PS_301-CG2 | 1.03 | 1.01 | 1.05 | 0.003 |
| PS_301-CG1 | 1.03 | 1.01 | 1.05 | 0.003 |
| PS_306-CG2 | 1.05 | 1.02 | 1.08 | 0.003 |
| PS_306-CG1 | 1.04 | 1.01 | 1.06 | 0.003 |
| PS_307-CG1 | 1.05 | 1.02 | 1.08 | 0.003 |

Association between CpG methylation levels and tumor grade determined by multivariable logistic regression analysis comparing low-grade (<G3) and high-grade (≥G3) RCC tumors, adjusted for age and sex. Odds ratios (ORs) are given per 1% increase in DNA methylation for individual CpG sites within LINC01354 (PS_301) and LINC01510 (PS_306 and PS_307). Tumor grade (low grade vs. high grade) served as the binary outcome variable. Corresponding 95% confidence intervals and Benjamini-Hochberg-adjusted p-values are shown. Age and sex were not significantly associated with tumor grade (data not shown). RCC, renal cell carcinoma; PS, pyrosequencing assay; CG, CpG site; OR, odds ratio; CI, confidence interval; *p*.adj, Benjamini-Hochberg-adjusted *p*-value; low grade, <G3; high grade, ≥G3.

# Supplemental Table S4

| CpG | Chr. | Pos. |
| --- | --- | --- |
| cg10811426 | chr1 | 234,667,461 |
| cg19856705 | chr1 | 234,667,500 |
| cg21546522 | chr1 | 234,667,549 |

Genomic localization of the CpG sites included in the *in silico* analysis of the TCGA-KIRC cohort. Note that cg21546522 was also part of the candidate differentially methylated region (DMR) identified in the present study. CpG, cytosine-phosphate-guanine site; Chr., chromosome; Pos., genomic position; TCGA-KIRC, The Cancer Genome Atlas Kidney Renal Clear Cell Carcinoma cohort; DMR, differentially methylated region.

# Supplemental Table S5

| Clin. param. | CpG | *p* | OR | Lower CI | Upper CI | *p*.adj | ΔM |
| --- | --- | --- | --- | --- | --- | --- | --- |
| Metastasis | cg10811426 | 0.000437 | 22.3 | 4.0 | 128.9 | 0.000655 | 0.094 |
|  | cg19856705 | 0.001707 | 10.4 | 2.4 | 45.3 | 0.001707 | 0.096 |
|  | cg21546522 | 0.000308 | 11.7 | 3.1 | 44.9 | 0.000655 | 0.120 |
| Lymph node metastasis | cg10811426 | 0.140155 | 0.4 | 0.1 | 1.4 | 0.171882 | -0.030 |
|  | cg19856705 | 0.171882 | 0.4 | 0.1 | 1.4 | 0.171882 | -0.032 |
|  | cg21546522 | 0.149819 | 0.4 | 0.1 | 1.3 | 0.171882 | -0.036 |
| Tumor stage | cg10811426 | 0.000317 | 14.1 | 3.4 | 61.4 | 0.000476 | 0.076 |
|  | cg19856705 | 0.001446 | 7.6 | 2.2 | 27.0 | 0.001446 | 0.076 |
|  | cg21546522 | 0.000069 | 11.1 | 3.5 | 37.6 | 0.000206 | 0.104 |
| Histological grade | cg10811426 | 0.000003 | 43.8 | 9.3 | 228.8 | 0.000010 | 0.099 |
|  | cg19856705 | 0.000027 | 20.4 | 5.2 | 88.3 | 0.000027 | 0.101 |
|  | cg21546522 | 0.000011 | 20.8 | 5.7 | 85.3 | 0.000016 | 0.116 |

TCGA-KIRC-derived data demonstrating associations between CpG methylation levels and clinicopathological parameters determined by multivariable logistic regression analysis adjusted for age and sex. Odds ratios (ORs) with corresponding 95% confidence intervals are shown for metastatic status (M0 vs. M+), lymph node metastasis (N0 vs. N+), tumor stage (≥pT3 vs. <pT3), and histological grade (≥G3 vs. <G3). Benjamini-Hochberg-adjusted p-values (p.adj) are shown. ΔM represents the mean difference in DNA methylation (*β*-values) between the compared groups. Clin. param., clinicopathological parameter; CpG, cytosine-phosphate-guanine site; OR, odds ratio; CI, confidence interval; *p*.adj, Benjamini-Hochberg-adjusted p-value; ΔM, mean difference in DNA methylation (*β*-values); TCGA-KIRC, The Cancer Genome Atlas Kidney Renal Clear Cell Carcinoma; M0, patients without distant metastasis; M+, patients with distant metastasis; N0, no lymph node metastasis; N+, lymph node metastasis.

# Supplemental Table S6

|  | Assay | *p* | HR | Lower CI | Upper CI |
| --- | --- | --- | --- | --- | --- |
| Univariable Cox regression | cg19856705 | 0.000001 | 3.43 | 2.09 | 5.63 |
|  | cg10811426 | 0.000015 | 2.45 | 1.63 | 3.67 |
|  | cg21546522 | 0.000000 | 3.93 | 2.48 | 6.24 |
| Multivariable Cox regression | cg19856705 | 0.001275 | 2.38 | 1.41 | 4.05 |
|  | cg10811426 | 0.262145 | 1.30 | 0.82 | 2.05 |
|  | cg21546522 | 0.006436 | 2.00 | 1.22 | 3.30 |

Results of univariable and multivariable Cox proportional hazards regression analyses assessing the association between CpG methylation levels and overall survival in the TCGA-KIRC cohort. Hazard ratios (HRs) with corresponding 95% confidence intervals are shown. CpG sites are listed in genomic order. CpG, cytosine-phosphate-guanine site; HR, hazard ratio; CI, confidence interval; TCGA-KIRC, The Cancer Genome Atlas Kidney Renal Clear Cell Carcinoma.

# Supplemental Table S7

| Tissue | Cases (TCGA-KIRC) | Gene | CpG site (*β*-value) | *R* | *p*.adj. |
| --- | --- | --- | --- | --- | --- |
| Tumor-adjacent normal tissue (adN) | 24 | LINC01354 | cg10811426 | -0.556 | 5.71*10^-3^ |
|  | 24 | LINC01354 | cg19856705 | -0.589 | 3.67*10^-3^ |
|  | 24 | LINC01354 | cg21546522 | -0.495 | 1.39*10^-2^ |
| Tumor tissue | 294 | LINC01354 | cg10811426 | -0.380 | 2.41*10^-11^ |
|  | 294 | LINC01354 | cg19856705 | -0.343 | 1.87*10^-9^ |
|  | 294 | LINC01354 | cg21546522 | -0.305 | 9.79*10^-8^ |
|  |  |  |  |  |  |

Results of correlation analysis evaluating the association between DNA methylation and gene expression to assess epigenetic silencing using the TCGA-KIRC cohort. DNA methylation of gene-associated CpG sites (*β*-values) was correlated with LINC01354 mRNA expression. adN, tumor-adjacent normal tissue; CpG, cytosine-phosphate-guanine site; TCGA-KIRC, The Cancer Genome Atlas Kidney Renal Clear Cell Carcinoma; R, Pearson correlation coefficient; p.adj, Benjamini-Hochberg-adjusted p-value.
